# COVID-19, Older People’s Alcohol Use and Engagement With Support: A Rapid Evidence Synthesis

**DOI:** 10.1093/geroni/igab046.1113

**Published:** 2021-12-17

**Authors:** Bethany Bareham, Eileen Kaner, Liam Spencer, Hannah O'Keefe, Barbara Hanratty

**Affiliations:** 1 Newcastle University, Newcastle upon Tyne, England, United Kingdom; 2 Newcastle University Population Health Sciences Institute, Newcastle upon Tyne, England, United Kingdom

## Abstract

COVID-19, and associated restrictions, have likely impacted older people’s alcohol use and related support needs, given disrupted routines and stress increase alcohol use in older populations. This rapid evidence synthesis aimed to examine older people’s (aged 50+) alcohol use, and engagement with alcohol support services during COVID-19. Seventy-six articles were identified through systematic database searches, reporting 63 survey, five qualitative, three pilot, and five hospital admission studies; of general and service-user populations of older drinkers. Data were drawn together through narrative synthesis. Alcohol use increased for up to 32% of older people, including service-users; particularly older women. Increased use was linked to anxiety, depression and emotional distress. Decreased use was more common in some older populations; particularly Mediterranean. Barriers such as web access and safe transport affected older service-users’ engagement with support. Support to address hazardous alcohol use amongst older people must be prioritised in wake of the pandemic.

